# Disseminated cytomegalovirus disease after bendamustine: a case report and analysis of circulating B- and T-cell subsets

**DOI:** 10.1186/s12879-019-4545-7

**Published:** 2019-10-22

**Authors:** Andrea Cona, Daniele Tesoro, Margherita Chiamenti, Esther Merlini, Daris Ferrari, Antonio Marti, Carla Codecà, Giuseppe Ancona, Camilla Tincati, Antonella d’Arminio Monforte, Giulia Marchetti

**Affiliations:** 1 Clinic of Infectious Diseases, Department of Health Sciences, ASST Santi Paolo e Carlo, University of Milan, Via di Rudinì 8, 20142 Milan, Italy; 2 Department of Diagnostics and Public Health, University of Verona, Gianbattista Rossi Hospital, Piazzale L.A. Scuro, 10, 37134 Verona, Italy; 3 Department of Medical Oncology, ASST Santi Paolo e Carlo, Milan, Italy; 4 U.O. Radiologia, Ospedale di Vizzolo Predabissi, Vizzolo Predabissi, Milan, Italy

**Keywords:** Bendamustine, Disseminated CMV, Lymphoma, B-cells, T-cells

## Abstract

**Background:**

Bendamustine, used for the treatment of indolent B-cell non-Hodgkin lymphoma and chronic lymphocytic leukemia, is known to cause prolonged myelosuppression and lymphocytopenia and has been associated with the risk of developing serious and fatal infections. While reports of localized CMV infections in asymptomatic patients exist, disseminated CMV disease has not been described.

**Case presentation:**

We report the first case of disseminated CMV infection in a 75-year-old male diagnosed with lymphoplasmacytic lymphoma/Waldenström macroglobulinemia with massive bone marrow infiltration. Despite 6-cycle R-bendamustine chemotherapy resulted in a good partial response, the patient developed persistent fever and severe weight loss. Analysis of cerebrospinal fluid and peripheral blood revealed the presence of CMV-DNA, while the fundus oculi examination revealed bilateral CMV retinitis. Treatment with induction and maintenance drugs was complicated by neutropenia and deterioration of renal function with electrolyte imbalance. From an immunological standpoint, we observed a profound imbalances in phenotype and function of B- and T-cell subsets, with a high proportion of circulating total, activated CD69+ and CD80+ B-cells, a low γ/δ T-cell frequency with a high proportion of CD69- and CD38-expressing cells, and hyperactivated/exhausted CD4+ and CD8+ T-cell phenotypes unable to face CMV challenge.

**Conclusions:**

We hereby describe a severe form of disseminated CMV disease after R-bendamustine treatment. Our observations strongly support the careful clinical monitoring of CMV reactivation/infection in oncologic patients undergoing this therapeutic regimen.

## Background

Bendamustine is a highly efficacious chemotherapeutic alkylating drug used as monotherapy or in combination with rituximab (R), for the treatment of indolent B-cell non-Hodgkin lymphoma and chronic lymphocytic leukemia.

It is known to cause prolonged myelosuppression and lymphocytopenia [1] and has thus been associated with the risk of developing serious and fatal infections [2], although a recent meta-analysis did not show a higher frequency of infections when using bendamustine compared with other alkylating drugs [3]. In particular, reports of localized CMV infections (i.e. retinitis, gastritis) as well as positive CMV antigenemia in asymptomatic patients [4–6] exist in the literature, yet disseminated CMV disease has not been described.

## Case presentation

We report the case of a patient who developed systemic CMV infection with encephalitis, retinitis, gastritis and colitis after treatment with R-bendamustine. The patient’s clinical history was collected from the electronic medical chart upon signed written informed consent, approved by our Ethics Committee (Helsinki Declaration).

A 75-year-old male was diagnosed with lymphoplasmacytic lymphoma/Waldenström macroglobulinemia with massive bone marrow infiltration in 2016. Past medical history was unremarkable, except for IgM monoclonal gammopathy since 1995.

A 6-cycle R-bendamustine chemotherapy resulted in a good partial response, defined as no extramedullary symptoms with a normal bone marrow biopsy and a reduced, but still detectable, monoclonal IgM protein.

**Fig. 1:**
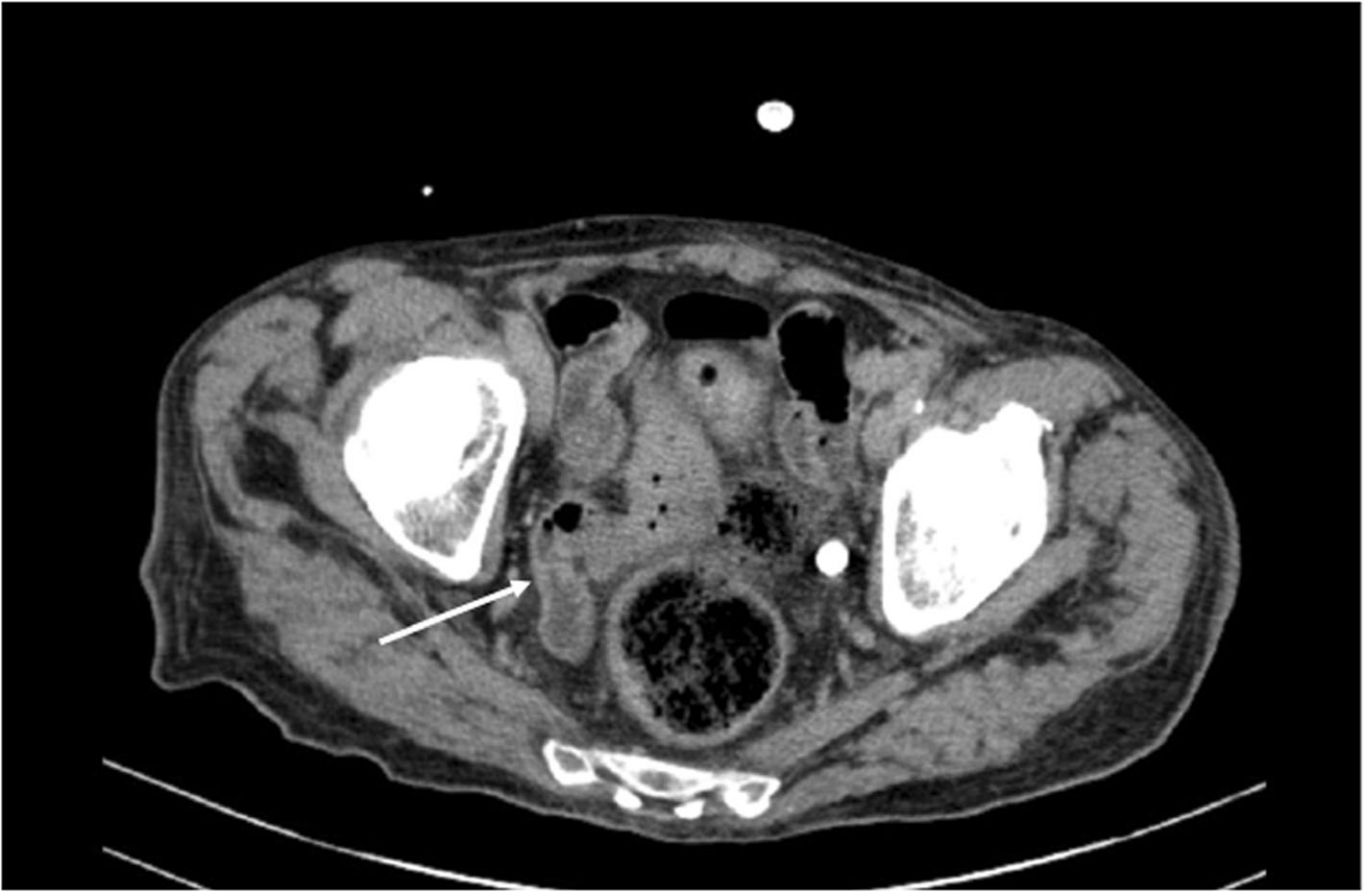
Abdominal CT scan. The arrow shows parietal thickening of the ileo-caecal region

Three months after treatment completion, the patient developed persistent fever and severe weight loss. Laboratory and radiological investigations excluded bacterial infections and extranodal lymphoma localizations. An abdominal CT scan showed parietal thickening of the ileo-caecal region (Fig. 1), and endoscopy revealed mucosal ulcers of both the oesophagus and the colon. Biopsies yielded CMV intracytoplasmic inclusions. HIV antibody testing was negative.

**Fig. 2:**
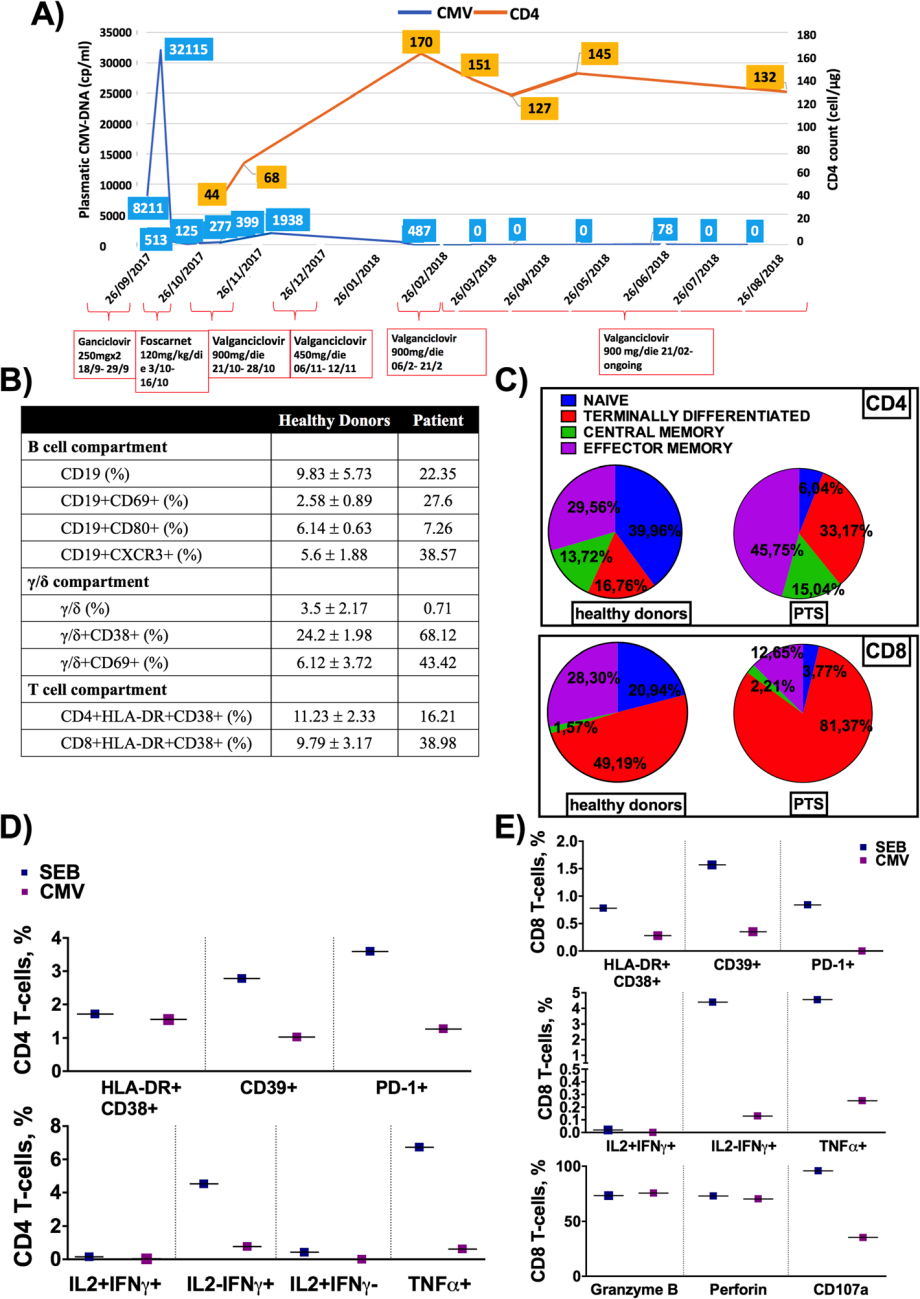
CMV-DNA and CD4+ T-cell count and characterization of B−/T-cell subsets. **a** Shows the trend of plasmatic CMV-DNA (cp/ml) and the trend of CD4 T-cell count (cell/μl). **b**-**e** shows flow cytometry results. Compared to healthy donors (*n* = 13; data presented as mean + SD value) the patient showed (i) higher proportion of circulating total B cells, CD69+, CD80+ and CXCR3+ B cells; (ii) lower frequency of circulating γ/δ T-cells, with higher proportion of CD69+ and CD38+ γ/δ T-cells; (iii) hyper-activated HLA-DR + CD38+ CD4 and CD8 T-cells (**b**). The study subject patient also featured an altered T-cell maturation profile, with massive loss of naïve (CCR7 + CD45RA+) and concomitant increase of terminally differentiated (CCR7-CD45RA+) CD4 and CD8 T-cells, with no substantial differences in central memory (CCR7 + CD45RA-) and effector memory (CCR7-CD45RA-) subpopulations compared to healthy donors (**c**). Staphylococcal Enterotoxin B (SEB_superantigen, blue) exposure resulted in high CD4+ T-cell expression of HLA-DR + CD38+ (1.71% vs 1.55), CD39+ (2.78% vs 1.03%) and PD-1+ (3.59% vs 1.27) compared to CMV stimulation (purple) (**d**; results are displayed after subtraction of “medium alone” condition). SEB, yet not CMV, also resulted in high CD8+ T-cell expression of HLA-DR + CD38+ (0.78% vs 0.28), CD39+ (1.57% vs 0.35%) and PD-1+ (0.84% vs 0) as well as IL2 + IFNγ+ (0.02% vs 0), IL2-IFNγ+ (4.40% vs 0.13%), TNFα+ (4.56% vs 0.25%) and CD107a (95.78% vs 35.43%) (**e**). Comparable levels of Granzyme B (73.2% vs 75.51%) and Perforin (72.85% vs 70.11%) were detected following both stinuli (**e**)

The patient underwent lumbar puncture for the onset of confusion with an abnormal EEG activity, consistent with an acute encephalopathy, despite aspecific *dura mater* enhancement upon brain MRI imaging, and was thus transferred to our clinical centre. He subsequently developed floaters and blurred vision; fundus oculi examination revealed bilateral CMV retinitis. CMV-DNA PCR was positive in both the peripheral blood (8200 cp/mL) and the CSF (34,500 cp/mL), thus a diagnosis of disseminated CMV infection was made with gastrointestinal, brain and ocular involvement. Induction treatment with ganciclovir (5 mg/Kg q12h) was started, soon after replaced by foscarnet (120 mg/Kg daily) due to the development of severe neutropenia on day 12. Foscarnet was suspended after 2 weeks of treatment due to the deterioration of renal function and electrolyte imbalances. After a 23-day cycle of induction therapy, despite residual plasmatic CMV-DNA (125 cp/mL), maintenance treatment with valganciclovir (900 mg/day) was started, subsequently reduced to 450 mg/day and finally stopped on day 15 because of neutropenia without complete suppression of CMV-viremia (CMV-DNA 399 cp/ml). T-lymphocyte immunephenotype performed 5 months after the last R-bendamustine cycle revealed severe CD4+ depletion (44 cells/μl, 16%), a CD8+ T-cell count of 158/μl (57%), and subversion of the CD4+/CD8+ ratio (0.28) (Fig. 2a).

Three months later the patient experienced reduced visual acuity and visual hallucinations. The *fundus oculi* examination revealed bilateral retinitis reactivation while no signs of encephalitis were found on brain-MRI. Hallucinations were accounted for as a side effect of levetiracetam, which was promptly discontinued. Plasmatic CMV-DNA resulted positive (487 cp/ml) and induction therapy with valganciclovir (900 mg q12h) was re-started. After 2 weeks, negativization of plasmatic CMV-DNA was observed and valganciclovir was reduced (900 mg/day). After the introduction of valganciclovir and discontinuation of levetiracetam, hallucinations resolved and visual acuity partially recovered as confirmed by the ophthalmologic evaluation that showed no signs of active lesions. At the beginning of suppressive maintenance therapy (9 months after chemotherapy) the CD4+, CD8+ T-cell counts and the CD4+/CD8+ T-cell ratio were 151 cells/μl, 578 cells/μl and 0.25 respectively (Fig. 2a).

Lymphoplasmacytic lymphoma/Waldenström macroglobulinemia is a B-cell lymphoma characterized by an infiltrate of heterogeneous B-cells and IgM hypersecretion [7]. Current therapeutic interventions target only lymphoplasmacytic cells [8], raising questions about the fate of the remaining B-cell subsets. We investigated the B-cell immune profile in this subject, finding a higher proportion of circulating total, activated CD69+ and CD80+ B-cells when compared to our in-house healthy control group (Fig. 2b). We also found a high proportion of B-cells expressing the chemokine receptor CXCR3 (Fig. 2b), known to regulate T-cell chemotaxis and to be expressed by B-cells in some subtypes of B-cell lymphoma [9], as further evidence of the profound imbalance within the B-lymphocyte compartment.

Given the development of disseminated CMV and the persistent CD4+ lymphopenia following a 6-cycle of R-bendamustine, we also sought to investigate T-cell immune-phenotype and function. We first assessed γ/δ T-cells, given their role in the immune response to CMV infection [10] and found a low γ/δ T-cell frequency with a high proportion of CD69- and CD38-expressing cells, suggesting a consumed, yet activated γ/δ compartment (Fig. 2b).

The assessment of CD4+ and CD8+ T-cells revealed a hyperactivated phenotype coupled with an altered distribution of memory and naïve subsets (Fig. 2b-c), compared to in-house healthy donors. Given that our patient displayed a hyperactivated T lymphocyte cell compartment, we next asked whether such generalized T-cell hyperactivation might also specifically affect the patient’s CMV-specific responses. Aiming to specifically dissect CMV-specific response, we therefore comparatively investigated functionally different CD4+/CD8+ T-cell subsets that have been demonstrated to play a central role in CMV-specific immune response [11].

Interestingly, the patient’s PBMC ex vivo challenge to CMV and bacterial stimuli revealed a different functional profile, with evidences of T-cell activation/exhaustion and IFN-γ/TNF-α release upon bacterial, but not CMV challenge (Fig. 2d-e). Likewise, our patient displayed a low CD107a release specifically after CMV challenge with no other differences in cytolytic activity (Fig. 2e), in all suggesting a selective impairment of CMV-specific immunity.

## Discussion and conclusions

To our knowledge, this is the first report of a disseminated CMV disease following treatment with bendamustine. While several case studies of localized CMV disease have been reported [12], no disseminated infection post-bendamustine has been described.

Aside from disease severity, the distinctive feature of our case resides in the entity and duration of the immunosuppression, known risk factor for CMV disease reactivation together with positive antibody CMV titres [13]. In particular, low total CD4+ T lymphocyte counts [2, 5], as well as steroid use [5], have been linked to overt clinical CMV disease following bendamustine treatment. In contrast to a report by Saito et al. who described resolution of lymphocytopenia approximately 7–9 months after treatment [1], in the present clinical case, CD4+ T-cells remained below 200/μl as long as 16 months after chemotherapy completion, implying a severe, long-lasting CD4+ depletion. Furthermore, bendamustine is known to affect the cytotoxic potential of CMV-specific CD8+ T-lymphocytes, in turn hampering the immune control over CMV [14]. Accordingly, our findings of a feeble CMV-specific response coupled with a hyperactivated, yet exhausted B- and T-cell phenotype with outgrowth of terminally-differentiated and effector memory at the disadvantage of naïve phenotypes, suggest functional exhaustion, possibly reflecting chronic viral antigenic exposure. Whether CMV infection further promotes the exhaustion of the immune system, or whether the impairment of the latter fuels CMV reactivation, remains to be clarified. Further, our data do not allow for the identification of a definite causal relationship between B/T cell immune pattern and the effects of bendamustine. Indeed, we were able to perform laboratory analyses 14 months following the start of bendamustine treatment and 1 year after the onset of CMV infection, with the patient on suppressive anti-CMV maintenance therapy exhibiting undetectable CMV viremia and a low CD4+ count (< 200 cells/μl). A detailed characterization of B/T lymphocyte homeostasis and function before and after bendamustine treatment should be encouraged to gain the broadest insight into the immune challenges of such therapies, to further assist clinical management of bendamustine-receiving patients.

From a clinical standpoint, patients with the above-mentioned characteristics are difficult to treat. Haematological side effects are common during treatment with ganciclovir, valganciclovir and foscarnet, possibly contributing to reduced life expectancy. Accordingly, we were forced to suspend the secondary prophylaxis despite a low level residual CMV viremia due to neutropenia, which contributed to the second disease reactivation.

In conclusion, we describe a severe form of disseminated CMV disease after bendamustine treatment; our findings support the careful clinical monitoring of CMV reactivation/infection in oncologic patients undergoing this therapeutic regimen. Specifically, monitoring should include: (i) CMV serology prior to the initiation of bendamustine therapy (ii) absolute and CD4+ T lymphocyte counts counts before, during and after bendamustine treatment and (iii) CMV viral load/antigenimia in patients who develop fever and/or clinical signs compatible with CMV infection [2–5, 13].

## Data Availability

All relevant data and material are included in this publication.

## Abbreviations

CMV: Cytomegalovirus
CSF: Cerebrospinal fluid
CT: Computed tomography
CXCR3: Chemokine receptor 3
EEG: Electroencephalogram
HIV: Human immunodeficiency virus
IFN-γ: Interferon-γ
IgM: Immunoglobulinn M
PBMC: Peripheral blood mononuclear cells
PCR: Polymerase chain reaction
R-bendamustine: Rituximab-bendamustine
TNF-α: Τumor necrosis factor-α
